# Reply to Lago, T.R.; Bolognani, F. Comment on “Nazarloo et al. Oxytocin, Vasopressin and Stress: A Hormetic Perspective. *Curr. Issues Mol. Biol.* 2025, *47*, 632”

**DOI:** 10.3390/cimb48020194

**Published:** 2026-02-10

**Authors:** Hans P. Nazarloo, Marcy A. Kingsbury, Hannah Lamont, Caitlin V. Dale, Parmida Nazarloo, John M. Davis, Eric C. Porges, Steven P. Cuffe, C. Sue Carter

**Affiliations:** 1 Department of Psychiatry, College of Medicine-Jacksonville, University of Florida, 580 West 8th St., Tower II, 6th Floor, Jacksonville, FL 32209, USA; steven.cuffe@jax.ufl.edu (S.P.C.); suecarterporges@gmail.com (C.S.C.); 2 Lurie Center for Autism, Mass General Research Institute, Harvard Medical School, Charlestown, MA 02129, USA; makingsbury@mgh.harvard.edu (M.A.K.); hhl23@gsbs.rutgers.edu (H.L.); 3 Southern Methodist University, Dallas, TX 75205, USA; cate@smu.edu; 4 Traumatic Stress Research Consortium, Kinsey Institute, Indiana University, Bloomington, IN 47405, USA; pnazarl@iu.edu; 5 Department of Psychiatry, University of Illinois at Chicago, Chicago, IL 60607, USA; davisjm@uic.edu; 6 Department of Clinical and Health Psychology, College of Public Health and Health Professions, University of Florida, Gainesville, FL 32611, USA; eporges@ufl.edu; 7 Center for Cognitive Aging and Memory, Evelyn F. and William L. McKnight Brain Institute, University of Florida, Gainesville, FL 32611, USA

We thank Lago and Bolognani [1] for their thoughtful and collegial engagement with our review, “Oxytocin, Vasopressin and Stress: A Hormetic Perspective” [2]. Their comment [1] provides valuable insights regarding V1a receptor (V1aR) involvement in stress regulation, the importance of threat predictability, and the translational potential of V1aR antagonists. These contributions meaningfully extend and refine the discussion presented in our original manuscript.

Our review proposed that vasopressin (VP) and oxytocin (OT) comprise a dynamic, biphasic regulatory system that orchestrates both catabolic and anabolic phases of stress response hormesis. Lago and Bolognani highlight several receptor-specific and clinical aspects that reinforce and clarify elements of this framework. We address these points below.

## 1. V1aR Contributions to HPA-Axis Regulation and Neuroendocrine Adaptation

We appreciate the authors’ emphasis on the role of V1aR in habituation to repeated stress. Their cited work demonstrates that the V1aR blockade impedes glucocorticoid habituation and alters patterns of neural activation in stress-related circuits [3,4]. These findings are consistent with the hormetic principles outlined in our review, in which adaptive responses depend on coordinated regulation across vasopressin pathways.

V1aR-expressing regions, including the bed nucleus of the stria terminalis (BNST), amygdala, and prefrontal networks, are involved in vigilance, affective modulation, and behavioral adaptation that are central to stress–response hormesis. These functions complement the established role of V1bR in pituitary ACTH release and support the integrative nature of VP signaling across stress phases.

## 2. Predictability of Threat and Its Relevance to Stress Pathophysiology

Lago and Bolognani underscore the importance of distinguishing predictable (fear) from unpredictable (anxiety) threat. Human startle research consistently shows that anxiety-potentiated startle (APS) is elevated across disorders such as panic disorder, posttraumatic stress disorder, and social anxiety disorder, whereas fear-potentiated startle (FPS) is often relatively preserved [5,6,7,8,9,10,11].

This distinction is both clinically and mechanistically relevant. Hormetic processes can follow both predictable and unpredictable stressors, and predictability alone may not be a prerequisite for resilience. Where it may matter most is in how long VP-driven anxiety persists and how readily the system shifts into OT-mediated recovery, which may depend more on the environment after the stressor (post-stressor context) than on the stressor itself. We suggest that the post-stressor environment, particularly the presence of social support, may be among the most important modulators of hormetic adaptation and resilience following both predictable and unpredictable stressors, as the presence of others can provide feelings of “safety” to facilitate hormetic restorative processes. Unpredictable threats tend to prolong vasopressin-mediated vigilance, impair habituation, and increase allostatic load, whereas predictable stressors may more reliably permit adaptive calibration of stress responses. Thus, the emphasis on threat predictability adds important nuance to the proposed VP-OT hormetic framework.

## 3. V1aR Antagonists: Promise and Clinical Interpretation

The Comment outlines a growing body of evidence suggesting that V1aR antagonists reduce anxiety-potentiated startle, modulate amygdala reactivity, and improve social functioning in both clinical and subclinical populations [12,13,14,15,16,17]. These findings are consistent with VP’s known involvement in social vigilance and threat processing.

While our original review did not aim to propose specific clinical interventions, these observations align with our conceptual model of VP-OT dynamics. V1aR antagonism may attenuate excessive or prolonged catabolic activation during uncertain threat, thereby facilitating a shift toward oxytocin-mediated recovery. Importantly, this interpretation does not imply suppression of adaptive stress responses, but rather the blunting of V1aR signaling that transitions to a maladaptive state due to excessive or sustained activation. Thus, partial modulation of V1aR signaling may constrain sustained hypervigilance while preserving sufficient VP-OT coupling to support recovery and resilience.

From a hormetic perspective, stress responses are beneficial when appropriately scaled in intensity and duration. Therapeutic effects of V1aR antagonism may therefore arise from limiting prolonged uncertainty-driven activation within limbic circuits such as the bed nucleus of the stria terminalis and extended amygdala, rather than abolishing stress responsiveness altogether. These receptor-targeted approaches may be particularly relevant for disorders characterized by heightened sensitivity to unpredictable threat.

## 4. Integration of V1aR, V1bR, and OTR Pathways in the Hormetic Stress Cycle

The receptor-specific analysis provided by Lago and Bolognani reinforces the view that VP-OT interactions function as a coordinated regulatory system in which V1bR contributes to acute activation of the HPA axis, V1aR modulates limbic threat appraisal, habituation, and social cognition, and OTR activation facilitates anti-inflammatory, parasympathetic, and restorative processes.

Together, these pathways support the biphasic organization of stress–response hormesis as a cycle of mobilization followed by recovery. The authors’ focused discussion of V1aR provides a valuable extension of this integrated framework.

## 5. Closing Remarks

We thank Lago and Bolognani for their collegial and insightful contribution. Their comment advances the discussion of vasopressin receptor dynamics and illustrates how neuroendocrine models of stress adaptation can inform translational research.

Taken together, these perspectives support a biphasic framework in which vasopressin and oxytocin coordinate mobilization and recovery across stress phases, with V1aR playing a potential role in calibrating responses to uncertainty and social context. This model yields testable predictions: specifically, that modulation of V1aR signaling should differentially affect habituation, threat predictability, and the transition from activation to recovery. Clarifying these mechanisms may help guide the development of targeted interventions for stress-related disorders characterized by dysregulated uncertainty processing.

## References

[1] Lago T.R., Bolognani F. (2026). Comment on Nazarloo et al. Oxytocin, Vasopressin and Stress: A Hormetic Perspective. *Curr. Issues Mol. Biol.* 2025, *47*, 632. Curr. Issues Mol. Biol..

[2] Nazarloo H.P., Kingsbury M.A., Lamont H., Dale C.V., Nazarloo P., Davis J.M., Porges E.C., Cuffe S.P., Carter C.S. (2025). Oxytocin, Vasopressin and Stress: A Hormetic Perspective. Curr. Issues Mol. Biol..

[3] Gray M., Innala L., Viau V. (2012). Central Vasopressin V1A Receptor Blockade Impedes Hypothalamic–Pituitary–Adrenal Ha-bituation to Repeated Restraint Stress Exposure in Adult Male Rats. Neuropsychopharmacology.

[4] Gray M., Innala L., Viau V. (2014). Central Vasopressin V1A Receptor Blockade Alters Patterns of Cellular Activation and Prevents Glucocorticoid Habituation to Repeated Restraint Stress Exposure. Int. J. Neuropsychopharm..

[5] Lago T.R., Brownstein M.J., Page E., Beydler E., Manbeck A., Beale A., Roberts C., Balderston N., Damiano E., Pineles S.L. (2021). The Novel Vasopressin Receptor (V1aR) Antagonist SRX246 Reduces Anxiety in an Experimental Model in Humans: A Randomized Proof-of-Concept Study. Psychopharmacology.

[6] Grillon C., Morgan C.A., Davis M., Southwick S.M. (1998). Effects of Experimental Context and Explicit Threat Cues on Acoustic Startle in Vietnam Veterans with Posttraumatic Stress Disorder. Biol. Psychiatry.

[7] Grillon C., Lissek S., Rabin S., McDowell D., Dvir S., Pine D.S. (2008). Increased Anxiety During Anticipation of Unpredictable but not Predictable Aversive Stimuli as a Psychophysiologic Marker of Panic Disorder. Am. J. Psychiatry.

[8] Grillon C., Pine D.S., Lissek S., Rabin S., Bonne O., Vythilingam M. (2009). Increased Anxiety During Anticipation of Unpre-dictable Aversive Stimuli in Posttraumatic Stress Disorder but Not in Generalized Anxiety Disorder. Biol. Psychiatry.

[9] Gorka S.M., Lieberman L., Shankman S.A., Phan K.L. (2017). Startle Potentiation to Uncertain Threat as a Psychophysiological Indicator of Fear-Based Psychopathology: An Examination Across Multiple Internalizing Disorders. J. Abnorm. Psychol..

[10] Grillon C., Chavis C., Covington M.F., Pine D.S. (2009). Two-Week Treatment with the Selective Serotonin Reuptake Inhibitor Citalopram Reduces Contextual Anxiety but not Cued Fear in Healthy Volunteers: A Fear-Potentiated Startle Study. Neuropsychopharmacology.

[11] Grillon C., Baas J.M.P., Pine D.S., Lissek S., Lawley M., Ellis V., Levine J. (2006). The Benzodiazepine Alprazolam Dissociates Contextual Fear from Cued Fear in Humans as Assessed by Fear-Potentiated Startle. Biol. Psychiatry.

[12] Hoge E.A., Armstrong C.H., Mete M., Oliva I., Lazar S.W., Lago T.R., Grillon C. (2024). Attenuation of Anxiety-Potentiated Startle After Treatment with Escitalopram or Mindfulness Meditation in Anxiety Disorders. Biol. Psychiatry.

[13] Albers H.E. (2015). Species, Sex and Individual Differences in the Vasotocin/Vasopressin System: Relationship to Neurochemical Signaling in the Social Behavior Neural Network. Front. Neuroendocr..

[14] Caldwell H.K. (2017). Oxytocin and Vasopressin: Powerful Regulators of Social Behavior. Neuroscientist.

[15] Meyer-Lindenberg A., Kolachana B., Gold B., Olsh A., Nicodemus K.K., Mattay V., Dean M., Weinberger D.R. (2009). Genetic Variants in AVPR1A Linked to Autism Predict Amygdala Activation and Personality Traits in Healthy Humans. Mol. Psychiatry.

[16] Lee R.J., Coccaro E.F., Cremers H., McCarron R., Lu S.-F., Brownstein M.J., Simon N.G. (2013). A Novel V1a Receptor Antagonist Blocks Vasopressin-Induced Changes in the CNS Response to Emotional Stimuli: An fMRI Study. Front. Syst. Neurosci..

[17] Bolognani F., Del Valle Rubido M., Squassante L., Wandel C., Derks M., Murtagh L., Sevigny J., Khwaja O., Umbricht D., Fontoura P. (2019). A Phase 2 Clinical Trial of a Vasopressin V1a Receptor Antagonist Shows Improved Adaptive Behaviors in Men with Autism Spectrum Disorder. Sci. Transl. Med..

